# Tanshinone IIA: A Promising Natural Cardioprotective Agent

**DOI:** 10.1155/2012/716459

**Published:** 2012-02-06

**Authors:** Qinghua Shang, Hao Xu, Li Huang

**Affiliations:** ^1^Graduate School, Beijing University of Chinese Medicine, Beijing 100029, China; ^2^National Integrative Medicine Center for Cardiovascular Diseases, China-Japan Friendship Hospital, Beijing 100029, China; ^3^Cardiovascular Center, Xiyuan Hospital, China Academy of Chinese Medical Sciences, Beijing 100091, China

## Abstract

Tanshinone IIA (Tan IIA) is a member of the major lipophilic components extracted from the root of *Salvia miltiorrhiza* Bunge, which is currently used in China and other neighboring countries to treat patients suffering from myocardial infarction (MI), angina pectoris, stroke, diabetes, sepsis, and other conditions. However, Tan IIA is not easy to be absorbed through intestinal pathway. To raise the bioavailability of the herb, sodium tanshinone IIA sulfonate (STS) was developed. This paper discussed the pharmacology of Tan IIA, STS, and their potential cardioprotective effects.

## 1. Introduction

*Salvia miltiorrhiza* Bunge (Danshen) belongs to the Labiatae family of the plant kingdom. It is considered to have the function of activating blood circulation and removing blood stasis, entering the “heart”, “pericardium”, and “liver” channels according to the theory of traditional Chinese medicine (TCM). Danshen has been widely used in oriental countries, especially China, to treat various circulatory disturbance-related diseases for its special pharmacological actions, including vasodilatation, anticoagulation, antiinflammation, and free radical scavenging. In recent years, traditional medicines have been playing more and more important roles in the maintenance of health, the prevention and treatment of diseases, as well as plant-based drug discovery [1, 2]. Although many practitioners are used to prescribing nature products, more and more doctors and researchers are fascinated in chemical compounds of *Salvia miltiorrhiza* Bunge.

**Figure 1:**
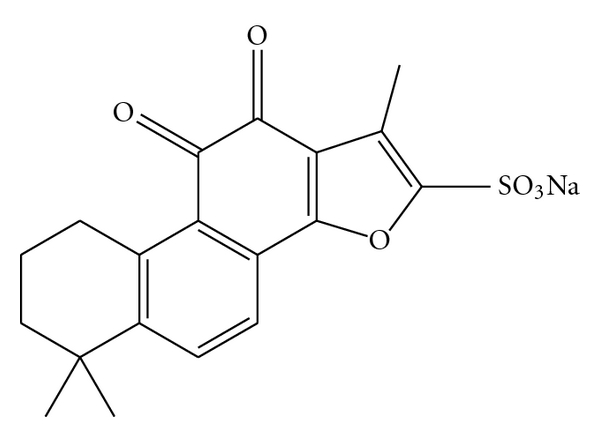
The chemical structure of STS.

There are two main active compounds: the lipophilic (Tanshinone I, IIA, IIB; cryptoTanshinone; other related compounds) and the hydrophilic (polyphenolic acids, danshensu, protocatechuic aldehyde, and protocatechuic acid). Tanshinone IIA (Tan IIA), which is a member of the major lipophilic components extracted from *Salvia miltiorrhiza* Bunge, has indicated significant therapeutic effects on various diseases* in vivo* and *in vitro*. Since Tan IIA is not easy to be absorbed through intestinal pathway, sodium tanshinone IIA sulfonate (STS) was developed to raise the bioavailability. The chemical structure of STS is shown in Figure 1. In this paper, the pharmacology of Tan IIA and STS in the treatment for cardiovascular diseases was reviewed.

## 2. Cardiovascular Pharmacology

### 2.1. Vasodilative Effect

Cheng et al. [3] demonstrated that Tan IIA produced a concentration-dependent relaxation in isolated spontaneously hypertensive rat (SHR) aortic rings precontracted with phenylephrine or potassium chloride (KCl) through ATP-sensitive K(+) channel to lower [Ca(2+)]*_i_*. Wu et al. [4] investigated the effects of Tan IIA on isolated rat coronary arteriole and the underlying mechanisms. The results showed that endothelium denudation, inhibition of nitric oxide synthase (NOS), inhibition of the cytochrome P450 epoxygenase, and blockade of the large conductance Ca(2+)-activated potassium channels (BKca) significantly decreased the vasodilation elicited by Tan IIA, which indicated that Tan IIA induces an endothelium-dependent vasodilation in coronary arterioles; nitric oxide (NO) and cytochrome P450 metabolites contribute to the vasodilation; activation of BKca channels plays an important role in the vasodilation. Kim et al. [5] concluded that topical Tan IIA increased both normalized arteriolar diameter and periarteriolar NO concentration in the two-kidney, one-clip renovascular hypertension model. N(G)-monomethyl-L-arginine inhibited Tan IIA-induced vasodilation. Tan IIA prevented the hypertension-induced reduction of endothelial NOS (eNOS) and increased eNOS expression to levels higher than sham-operated control. Topical Tan IIA increased normalized arteriolar diameter more in the cremaster muscle of control mice than that in cremasters of eNOS knockout mice. In ECV-304 cells transfected with eNOS-green fluorescent protein, Tan IIA significantly increased eNOS protein expression and eNOS phosphorylation. The results also indicated that eNOS stimulation was one mechanism by which Tan IIA induced vasodilation and reduces blood pressure.

### 2.2. Inhibition of Left Ventricular Hypertrophy

Accumulative studies had demonstrated that Tan IIA could inhibit left ventricular hypertrophy (LVH) with different mechanisms. Overwhelming postloading is a main factor promoting LVH, therefore controlling hypertension is very important in LVH inhibition. Tan IIA has shown vasodilatation effect through adenosine triphosphate (ATP)-sensitive K(+) channel to lower the concentration of Ca^2+^ in myocytes, regulate the condition of hypertension, and inhibit the formation of hypertrophy [3]. Tan IIA could also prevent LVH through inhibiting angiotensin receptor (ATR) expression or blocking free Ca^2+^ influx in rats with hypertrophic myocardium caused by abdominal aorta constriction, and its effect on lowering hypertension had no significant difference compared with Valsartan [6, 7]. Additionally, Tan IIA has been reported to block the transforming growth factor (TGF) beta1/Smads signal pathway and inhibit the formation of myocardial hypertrophy [7], attenuate enhanced collagen type I expression and collagen synthesis as well as depressed matrix metalloproteinase-1 (MMP-1) expression and activity by angiotensin II (Ang II) [8]. Furthermore, Tan IIA depressed the intracellular generation of reactive oxygen species (ROS), nicotinamide adenine dinucleotide phosphate (NADPH) oxidase activity, and subunit p47 (phox) expression, which are the factors inducing LVH. Fang et al. [9] concluded that Tan IIA conferred its beneficial effects on the collagen metabolism probably through its regulation of transcript levels of the MMPs/tissue inhibitor of metalloproteinases (TIMPs) balance. Although the balance regulation had no difference as compared with Valsartan, Tan IIA showed slight improvement in attenuating cardiac dysfunction. At least three studies assessed proto-oncogene c-fos mRNA expression of cardiocytes when investigated the mechanism of Tan IIA, indicating that Tan IIA could prevent LVH induced by Ang II, which might be related to its inhibition of proto-oncogene expression [10–12]. Similar conclusion was drawn by Tu et al. [13] that Tan IIA had the definite function in preventing LVH by its action on the protein kinase B (PKB/Akt) signaling pathway, which could regulate the expression of proto-oncogene c-fos. Two *in vitro* studies [14, 15] had demonstrated that Tan IIA dose-dependently inhibited the increment of the total protein level induced by Ang II and the p-extracellular signal regulatory kinase (ERK)1/2 expression stimulated by Ang II, which indicated the mechanism that Tan IIA inhibited the myocardial cell hypertrophy induced by Ang II may be associated with the inhibition of p-ERK1/2.

### 2.3. Restraining Smooth Muscle Cell Proliferation and Intimal Hyperplasia

Tan IIA could significantly decrease intimal thickening, suppress cell proliferation and migration, inhibit the expression of various growth factors, induce the differentiation, maturity, and apoptosis of the vascular smooth muscle cell (VSMC), and ameliorate the function and condition of vascular smooth muscle [16]. Since there are so many beneficial effects on VSMC, Tan II is playing an important role in the treatment of arteriosclerosis, restenosis after angioplasty or stenting, brain arteriovenous malformations, and pulmonary hypertension. However, the mechanism of Tan II has not been very clear. Various studies [17–21] had found that Tan IIA could suppress cell proliferation and BrdU incorporation into DNA, block cell cycle in G0/G1 phase, and inhibit ERK1/2 phosphorylation and c-fos expression. Initial proliferation might be inhibited by blocking mitogen-activated protein kinase (MAPK) signaling pathway and downregulating c-fos expression. Pan et al. [22] demonstrated that Tan IIA could significantly inhibit the proliferation of VSMCs in a dose-dependent manner, and the mechanism might be related to the downregulation of calponin (CaN) activities and the inhibition on calcineurin mRNA and proliferating cell nuclear antigen (PCNA) expressions. Jin et al. [23] illuminated that Tan IIA exhibited multiple effects on inhibiting human aortic SMCs migration, the mechanisms of which might inhibit IkappaBalpha phosphorylation and p65 nuclear translocation through inhibition of Akt phosphorylation, suppress tumor necrosis factor-alpha (TNF-*α*)-induced ERK and c-jun phosphorylation, and block nuclear factor-kappaB (NF-*κ*B) and activator protein-1 (AP-1) DNA-binding; all these factors played important roles in human aortic SMCs migration.

### 2.4. Attenuation of Atherosclerosis

In addition to VSMC proliferation and intimal hyperplasia, injury of vascular endothelium, lipid deposition, oxidative stress, and inflammatory reaction also play important roles in the formation and progression of atherosclerosis. Endothelial cells can secrete two kinds of substances with opposite functions, one can induce VSMC apoptosis (such as NO), and the other can inhibit VSMC apoptosis (such as endothelin-1 (ET-1), Ang II, and growth factors). Unbalance between them decides whether endothelium is injured. NO is the key factor in signal transduction, it can relax blood vessels and activate genes relative to VSMC apoptosis. Li et al. [24] and Huang et al. [25] concluded that Tan IIA could inhibit the negative effect of Ang II on NO production and eNOS expression in porcine aortic endothelial cells. Another *in vitro* study showed that Tan II might inhibit ET-1 production and cell apoptosis, inducing protective effect on vessel endothelium [26]. Tan IIA could also reduce plaque area in endothelium, decrease lipid deposition, and significantly inhibit the formation of atherosclerosis, although the level of total cholesterol (TC), triglyceride (TG), low-density lipoprotein cholesterol (LDL-C), and high-density lipoprotein cholesterol (HDL-C) in serum had not been changed by Tan IIA [27].

To verify the antioxidant effect on atherosclerosis formation, at least five experiments [28–32] had been established. Tang et al. [28] demonstrated that Tan IIA could attenuate atherosclerotic lesion in apolipoprotein E (apoE)(−/−) mice, which might be attributed to its properties of both antioxidation and downregulation of scavenger receptors. Furthermore, antagonism of peroxisome proliferators-activated receptor gamma (PPAR*γ*) might be involved in the downregulation of CD36 by Tan IIA. Fang et al. [29] found that the superoxide dismutase (SOD) activity was significantly increased while the level of malondialdehyde (MDA) was decreased in Tan IIA group, which showed that antioxidant effect of Tan IIA might be a potential mechanism involved in antiatherosclerosis. Tang et al. [30] suggested that Tan II A significantly attenuated the atherosclerosis in rat model, which might be attributed to its inhibition of oxidized low density lipoprotein (oxLDL) production, independent of the serum levels of lipids, calcium, and 25-OH Vitamin D. Increasing of Cu/Zn SOD activity as well as mRNA and protein expression by Tan IIA might protect LDL against oxidation induced by superoxide anion in vessel. Active oxygen free radical is a major factor inducing endothelial injury; hydrogen peroxide can promote the formation of free radicals, which can penetrate cell membrane, combine with Fe^2+^ or Cu^2+^, induce lipid peroxidation, and lead to endothelium injury and formation of atherosclerosis finally. Lin et al. [31, 32] indicated that Tan IIA could protect ECV-304 cell damage induced by hydrogen peroxide through its anti-oxidant effect and CD40 anti-inflammatory approach.

Inflammatory effect can induce endothelium injury, foam-cell appearance, and leukocytes adhesion, all of which play important roles in the formation of atherosclerosis. At least two studies [29, 33] had verified that Tan IIA could decrease inflammatory effect and attenuate atherosclerosis of vessels. Fang et al. [29] indicated that expression reduction of CD40 and MMP-2 activity might be the potential mechanisms of antiatherosclerosis effect of Tan IIA. Fang et al. [33] demonstrated that Tan IIA could dose-dependently inhibit atherosclerotic lesion through downregulation of protein expression and activities of MMP-2 and MMP-9 as well as serum VCAM-1 and interleukin (IL)-1*β* in rabbits fed high-fat diet.

Platelet activation and aggregation can accelerate the formation of atherosclerosis. Jiang et al. [34] indicated that Tan IIA could inhibit the increasing P-selectin expression of thrombin-activated platelets in a concentration-dependent manner, which may also be a mechanism of Tan IIA to inhibit atherosclerosis.

### 2.5. Lipid-Lowering Effect

Kang et al. [35] demonstrated that human HepG2 cells treated with Tan IIA for 24 h exerted a dose-dependent inhibitory effect on apolipoprotein B (apoB) secretion together with TG. However, another secretory protein, albumin, was unaffected by Tan-IIA treatment, indicating that the effect of Tan IIA is specific for apoB secretion. Tan IIA decreased the transcription level of microsomal TG transfer protein gene, suggesting that lipoprotein assembly is likely to be involved in the inhibited ApoB secretion. Gong et al. [36] reported that Tan IIA inhibited 3T3-L1 preadipocyte differentiation and transcriptional activities of full-length PPAR*γ* and PPAR*γ* ligand-binding domains. The effects of Tan IIA are mediated through its property as a natural antagonist of PPAR*γ*. Tan IIA treatment reduced adipose mass and body weight, improved glucose tolerance, and lowered the LDL/HDL ratio without changing the food intake in a high-fat-diet-induced obese animal model.

### 2.6. Inhibitory Effect on the Inflammatory Responses

Various studies demonstrated that inflammatory response was involved in the process of myocardial infarction (MI), endothelium injury, atherosclerosis, and cardiovascular hypertrophy [23, 29, 33, 37, 38], which have been mostly introduced in the former paragraphs. However, mechanisms underlying this effect have not been fully understood. NF-*κ*B activation by NF-*κ*B-inducing kinase (NIK)-lkappaB alpha kinase (IKK) pathway and MAPKs pathway is known to be involved in the inflammatory response. Jang et al. [39] determined the inhibitory effect of Tan IIA on the activation of NF-*κ*B and IkappaB alpha phosphorylation and also examined phosphorylation of NIK and IKK as well as the activation of MAPKs such as p38 MAPK (p38), ERK1/2, and c-Jun N-terminal kinase (JNK) in RAW 264.7 cells stimulated with Lipopolysaccharides (LPS). The result suggested that Tan IIA might inhibit LPS-induced lkappaB alpha degradation and NF-*κ*B activation via suppression of the NIK-IKK pathway as well as the MAPKs (p38, ERK1/2 and JNK) pathway in RAW264.7 cells, and these properties might provide a potential mechanism which could explain the anti-inflammatory activity of Tan IIA. Another* in vitro* study [40] suggested that Tan IIA had a similar structure with 17 beta-estradiol (E2) and the result indicated Tan IIA exerted anti-inflammatory effects by inhibition of inducible NOS (iNOS) gene expression and NO production, as well as inhibition of inflammatory cytokine (IL-1*β*, IL-6, and TNF-*α*) expression via estrogen receptor-dependent pathway. Therefore, it could serve as a potential selective estrogen receptor modulator (SERM) to treat inflammation-associated neurodegenerative and cardiovascular diseases without increasing the risk of breast cancer. Similar results were demonstrated by other studies [41, 42].

### 2.7. Antioxidant Effect

Oxidation reaction was involved in various pathological mechanisms, inducing different diseases including MI, angina pectoris, and restenosis after PCI, LVH, and so on. Tan IIA can inhibit these reactions, which have been mentioned in the above experiments [8, 28–32]. To test the hypothesis that Tan IIA can alter the expression and/or activity of specific antioxidant enzymes to prevent cells from oxidant damage, at least three experiments [38, 43, 44] were conducted and demonstrated that the cell protective effect of Tan IIA was mediated primarily by induction of glutathione peroxidase (GPx) gene expression and activity, as well as other antioxidant enzyme activities in the heart. At least four experiments [44–47] indicated that Tan IIA could scavenge the free radicals produced in the superoxide approach, which might be one of the important mechanisms in myocardiocyte damage. Other studies [30, 48] suggested that Tan IIA significantly attenuates myocardiocyte or vasculocyte damage, which might be attributed to its inhibition of ox-LDL production.

### 2.8. Antiplatelet, Anticoagulant, and Antithrombotic Effect

Tan IIA can decrease the blood viscosity obviously, inhibit the activation of thrombin, and promote fibrin degradation; it can inhibit the function of platelets and the formation of thrombus. Li et al. [49] showed that Tan IIA could significantly decrease the platelet number, with efficacy similar to aspirin. Jiang et al. [34] also found that Tan IIA could reduce the number of blood platelets by inhibiting P-selectin expression in a concentration-dependent manner. Li et al. [50] demonstrated that Tan IIA could inhibit the thrombus formation and platelet aggression in *in vivo* study, and it exerted more significant effect on antiplatelet than anticoagulation.

To investigate the effects of Tan IIA on procoagulant activity (PCA) of human ECV304 cells induced by acute promyelocytic leukemia cell line NB4 cells, Zhang et al. [51] showed that the conditional media of NB4 cells treated with Tan IIA (Tan IIA-NB4-CM) can increase the levels of PCA and tissue factor (TF) activity of ECV304 cells through some unidentified factor; however, Tan IIA can obviously decrease the PCA and TF activity of ECV304 cells induced by Tan IIA-NB4-CM.

CD41 and CD62p are two of the most important inflammatory factors, which can induce platelets aggregation and promote blood coagulation. Jia et al. [52] found that Tan IIA could decrease the expression of CD41 and CD62p, which might inhibit platelet aggregation and blood coagulation.

### 2.9. Antiarrhythmia Effect

Jia et al. [52] indicated that Tan IIA could decrease the expression of adhesion molecule in blood platelet to prevent arrhythmia. In addition, high-conductance Ca^2+^-activated K^+^ channels (BK_Ca_) in vascular smooth muscle also play important roles in controlling the vascular tone by determining the level of membrane potential and Ca^2+^ influx through voltage-gated Ca^2+^ channels. Agents that can alter the activity of Ca^2+^ channels or BK_Ca_ thus affect the vascular tone in both physiological and pathological conditions. Experiments [53, 54] showed that Tan IIA could block L-type Ca^2+^ channel, decrease concentration of intracellular Ca^2+^, ameliorate calcium overload in myocardiocytes, and prevent or even treat arrhythmia finally. Except for Ca^2+^ and K^+^, microRNA-1 (miR-1) level is also one of the important factors in ischemic arrhythmia. Shan et al. [55] indicated downregulation of miR-1 and consequent recovery of Kir2.1 might account partially for the efficacy of Tan IIA in suppressing ischemic arrhythmia and cardiac mortality. These findings support the proposal that miR-1 could be a potential therapeutic target for the prevention of ischemic arrhythmias. On gene level, Sun et al. [56] indicated that Tan IIA could activate human cardiac KCNQ1/KCNE1 potassium channels (*I*_Ks_) in HEK 293 cell directly and specifically through affecting the channels' kinetics, which would be a promising therapeutic medicine in arrhythmia.

### 2.10. Antimyocardial Hypoxia

Reducing oxygen consumption and increasing the tolerance in hypoxygen of myocardiocytes are beneficial to coronary heart disease. Huang et al. [57] detected the left ventricle end diastole pressure (LVEDP) after ligating coronary artery of dogs, the result indicated Tan IIA could decrease LVEDP and heart volume and reduce myocardial oxygen consumption. Shao et al. [58] demonstrated that the activation of ATP enzyme in myocardium was decreased in patient with hyperthyroidism; however, Tan IIA could protect it and increase the tolerance in hypoxygen of the myocardiocytes. Various studies [4, 59, 60] have suggested that Tan IIA might dilate coronary artery, inhibit vascular contraction, increase coronary blood flow and reduce oxygen consumption of myocardiocytes with different mechanisms. Sun et al. [61] suggested that Tan IIA could decrease intracellular calcium overload and K+ outflow, inhibit Na+ inflow, keep the balance of membrane potential, and therefore protect myocardiocyte in hypoxia.

### 2.11. Reduction of Myocardial Infarct Size

Tan IIA can dilate coronary artery and increase coronary blood flow, which is beneficial for reducing MI size. Various experiments [62–65] have demonstrated that Tan IIA might recover cardiac function and reduce MI size significantly with different mechanisms. Zhang et al. [62] indicated that the possible mechanism responsible for the effect of Tan IIA was associated with the phosphatidylinositol 3-kinase (PI3K/Akt)-dependent pathway, which was accompanied with decreased cardiac apoptosis and inflammation. In addition, Tan IIA was found to reduce MI size by 53.14 ± 22.79% as compared to that in the saline control, simultaneously, and significantly prolonged the survival of cultured human saphenous vein endothelial cells rather than human ventricular myocytes *in vitro* (these cells were separately exposed to xanthine oxidase (XO)-generated oxyradicals), which may suggest that Tan IIA could reduce MI size through prolonging survival of endothelial cells [63]. Xu et al. [64] have assessed the effect of Tan IIA on endothelial cells of MI in rats, and they suggested that Tan IIA could reduce MI size and myocardial ischemia injury through promoting angiogenesis and upregulating vascular endothelial growth factor (VEGF) expression. Jiang et al. [65] found that Tan IIA might establish extensive collateral circulation and increase blood flow in ischemic area.

### 2.12. Inhibiting Ischemia Reperfusion Injury

Ischemia reperfusion (IR) exerts disturbance of microcirculation and leads to many diseases, including myocardial stunning and reperfusion arrhythmia. Production of oxygen free radicals, calcium overload in myocytes, endothelial cell injury, adhesion of leukocyte, energy supply reduction, mitochondrial damage, and myocardiocytes apoptosis are considered to be involved in this process. Tan IIA can inhibit the activation of proteases and ameliorate calcium overload in myocytes, which have been introduced in the previous paragraph [53, 54, 61]. In addition, Tan IIA can increase the SOD content in the injured myocytes, decrease the MDA concentration, and influence electron transfer reaction in mitochondria, thus scavenge the free acids, reduce the lipid peroxidation, and protect myocytes and vascular endothelial cells in the IR process [45–47]. ET, which can induce constriction of the vessel, increases significantly in IR, and Tan IIA can inhibit the production and release of ET, promote secretion of NO, and decrease IR injury of the heart [24–26]. Jiang et al. [34] indicated that Tan IIA could inhibit HL-60 cell adhesion to human umbilical vein endothelial cells through concentration dependently inhibiting TNF-alpha and ameliorate microcirculation disturbance. Fu et al. [46] suggested that Tan IIA might markedly inhibit H_2_O_2_-induced oxidation *in vitro*, significantly inhibit IR-induced cardiomyocyte apoptosis by attenuating morphological changes and reducing the percentage of terminal transferase dUTP nick end-labeling (TUNEL)-positive myocytes and caspase-3 cleavage, as well as ameliorate IR injury by upregulating Bcl-2/Bax ratio.

## 3. Final Comments

In the beginning of 21st century, we are facing serious challenges of cardiovascular diseases (CVDs). Although it is becoming less lethal, CVD prevalence is incessantly increasing and it is still the most common cause of death. As a representative of complementary and alternative medicines, TCM has a history of thousands of years and has made great contributions to the health and wellbeing of the people and to the maintenance and growth of the population. It provides us with great treasure of herbal medicines or natural products, which can be served as lead compound or new drug candidates in the battle against CVDs.

Herbal medicines with the function of activating blood circulation (ABC) have been investigated extensively and made remarkable achievements in recent years [66, 67]. *Salvia miltiorrhiza* Bunge is the most common used ABC herb in China for treating CVDs and other circulatory disturbance-related diseases. Tan IIA, which is a member of the major lipophilic components extracted from *Salvia miltiorrhiza* Bunge, has indicated significant therapeutic effects and multiple pharmacological actions including vasodilative, antithrombotic, anti-inflammation, antioxidant, anti-ischemia, antiarrhythmia, antihyperplasia, antiatherosclerosis, and lipid-lowering effect. Clearly, Tan IIA appears to be a promising natural cardioprotective agent. Further research is warranted to translate these beneficial effects into clinical practice and definitely address the mechanisms of its multitarget actions.
